# CTLA-4 suppresses hapten-induced contact hypersensitivity in atopic dermatitis model mice

**DOI:** 10.1038/s41598-023-35139-y

**Published:** 2023-05-16

**Authors:** Hiroe Tetsu, Kanako Nakayama, Taku Nishijo, Takuo Yuki, Masaaki Miyazawa

**Affiliations:** Safety Science Research Laboratories, Kao Corporation, 2606 Akabane, Ichikai, Haga, Tochigi, 321-3497 Japan

**Keywords:** Inflammation, Lymphocytes

## Abstract

Atopic dermatitis (AD) patients with skin barrier dysfunction are considered to be at a higher risk of allergic contact dermatitis (ACD), although previous studies showed that attenuated ACD responses to strong sensitizers in AD patients compared to healthy controls. However, the mechanisms of ACD response attenuation in AD patients are unclear. Therefore, using the contact hypersensitivity (CHS) mouse model, this study explored the differences in CHS responses to hapten sensitization between NC/Nga mice with or without AD induction (i.e., non-AD and AD mice, respectively). In this study, ear swelling and hapten-specific T cell proliferation were significantly lower in AD than in non-AD mice. Moreover, we examined the T cells expressing cytotoxic T lymphocyte antigen-4 (CTLA-4), which is known to suppress T cell activation, and found a higher frequency of CTLA-4^+^ regulatory T cells in draining lymph node cells in AD than in non-AD mice. Furthermore, the blockade of CTLA-4 using a monoclonal antibody eliminated the difference in ear swelling between non-AD and AD mice. These findings suggested that CTLA-4^+^T cells may contribute to suppressing the CHS responses in AD mice.

## Introduction

Atopic dermatitis (AD) and allergic contact dermatitis (ACD) are common inflammatory skin diseases. The impaired skin barrier function and immune system abnormalities contribute to the pathogenesis of AD, a type 2 inflammatory disease^1^. ACD is a T cell-mediated delayed type-hypersensitivity reaction caused by contact and penetration of low molecular weight chemicals called haptens into the skin^2^. Skin barrier dysfunction in AD patients may increase their susceptibility to ACD sensitization because of the increased allergen penetration^3^.

Several studies on the development of ACD in AD patients have shown that the susceptibility of sensitization to strong sensitizers may be lower in AD patients than in healthy controls^4–6^. A previous epidemiologic study found that the rate of positive patch test results against strong sensitizers was similar in AD patients and healthy controls^4^. In addition, clinical experiments using 2,4-dinitrochlorobenzene (DNCB) showed that ACD responses to DNCB were significantly lower, and Th1 cell proliferation was lower in the AD patients^5,6^. As such, it can be inferred that a mechanism of the attenuated Th1 response is at work during ACD in AD patients.

The mechanism by cytotoxic T lymphocyte antigen-4 (CTLA-4; CD152) is known as a regulatory mechanism that inhibits T cell activation and proliferation^7^. CTLA-4 is a receptor expressed on activated T cells and regulatory T (Treg) cells and can bind to the B7 family molecules CD80 (B7-1) and CD86 (B7-2), ligands present on antigen-presenting cells (APCs)^8,9^. Actually, contact hypersensitivity (CHS) mouse model that is useful to elucidate the mechanisms of ACD suggested that CTLA-4^+^T cells can suppress antigen-specific T cell proliferation during CHS response^10^.

AD patients have higher expression levels and percentages of CTLA-4^+^T cells in peripheral blood than the healthy controls^11,12^, suggesting the higher suppressive potential of CTLA-4^+^T cells in AD patients. However, it is unclear whether CTLA-4^+^T cells play a role in the attenuated Th1 response in AD patients.

As previous studies have not revealed the mechanism of the attenuated Th1 response in AD patients, the contribution of the CTLA-4^+^T cells to the mechanism remains unclear. Moreover, although mouse models help elucidate the mechanism, no AD mice model exhibits an attenuated CHS response similar to the one in humans^13,14^. Therefore, using an AD mouse model, this study focused on CTLA-4^+^T cells to elucidate the mechanism of attenuated Th1 response in AD patients. We first examined the CHS response to 2,4-dinitrofluorobenzene (DNFB) in NC/Nga mice, an AD mouse model that is highly homologous to AD patients^15^. After that, antigen-specific T cells and cytokine production in the draining lymph nodes (LNs) were analyzed, and the contribution of CTLA-4^+^T cells in CHS response was examined using an anti-CTLA-4 monoclonal antibody (mAb).

## Results

### CHS response attenuated in AD mice

**Figure 1:**
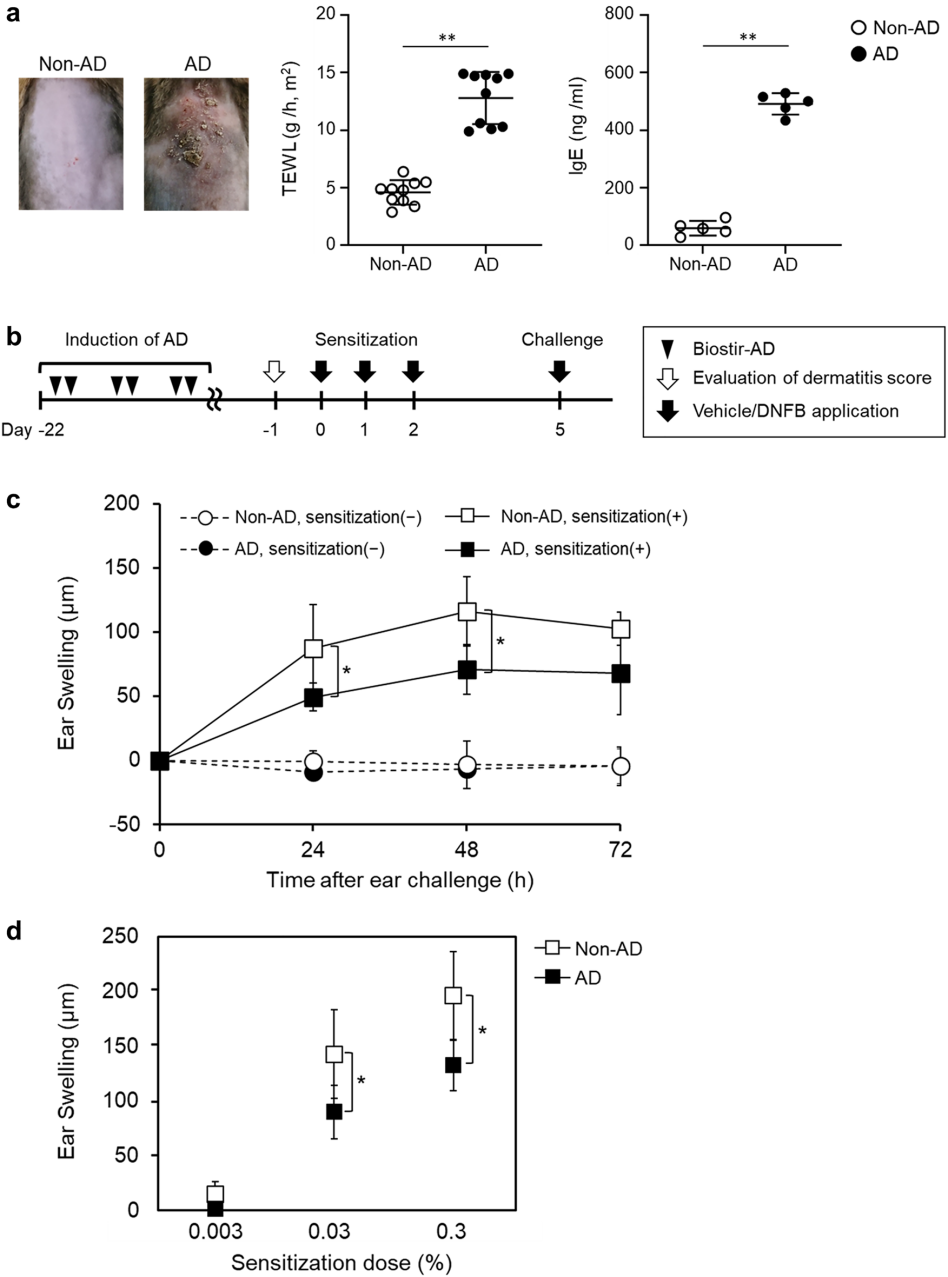
Decreased CHS response induced by DNFB in AD model mice. AD-like skin lesions in NC/Nga mice were induced by Biostir-AD. (**a**) Features of the back skin after AD induction. Transepidermal water loss (TEWL) through dorsal skin. Total serum IgE concentrations of non-AD and AD mice were measured by ELISA. (**b**) CHS responses were induced, as shown in the scheme. (**c**) Mice were sensitized with or without 0.3% DNFB on the dorsal skin, and ear swelling was measured after the challenge. Data represents the change in ear thickness at 24, 48 and 72 h. (**d**) Mice were sensitized with DNFB at the following concentrations: 0.003%, 0.03%, and 0.3%. Data represents the change in ear thickness at 48 h. Data were expressed as the mean ± *SD* (n = 5, TEWL data; n = 10) and represented three independent experiments with similar findings. *p < 0.05, **p < 0.01 between indicated groups.

First, we investigated whether the CHS responses of NC/Nga mice with AD induction (AD mice) differed from those without AD induction (non-AD mice) using a DNFB-induced CHS model. To compare the CHS responses between non-AD and AD mice, AD-like skin lesions were induced in NC/Nga mice. In the dorsal skin of AD mice, AD-like skin lesions, such as swelling, dryness, and erythema were observed (Fig. 1a). Trans-epidermal water loss (TEWL) of AD-like skin lesion and total serum immunoglobulin E (IgE) levels were significantly higher (TEWL; *p* = 0.00003, IgE; *p* = 0.00028) in AD than that in non-AD mice (Fig. 1a). Then, AD and non-AD mice were sensitized with 0.3% DNFB applied onto the shaved dorsal skin on days 0–2. Subsequently, the skin of the ear was challenged on day 5 with 0.3% DNFB (Fig. 1b). CHS response was assessed by the ear swelling that was induced by DNFB sensitization. Ear swelling at 24 and 48 h was significantly lower (24 h; *p* = 0.04, 48 h; *p* = 0.02) in DNFB-sensitized AD mice than in DNFB-sensitized non-AD mice (Fig. 1c). Moreover, the results of 0.03% DNFB-sensitized mice at 48 h after the challenge were similar to those of 0.3% DNFB-sensitized mice (0.03% DNFB; *p* = 0.04, 0.3% DNFB; *p* = 0.02). In contrast, ear swelling was not observed in either non-AD or AD mice sensitized with 0.003% DNFB (Fig. 1d). These data indicated that the CHS responses were suppressed in AD mice, regardless of the concentration.

### Reduced DNFB-specific T cell proliferation and IFN-γ production in the draining LNs of sensitized AD mice

**Figure 2:**
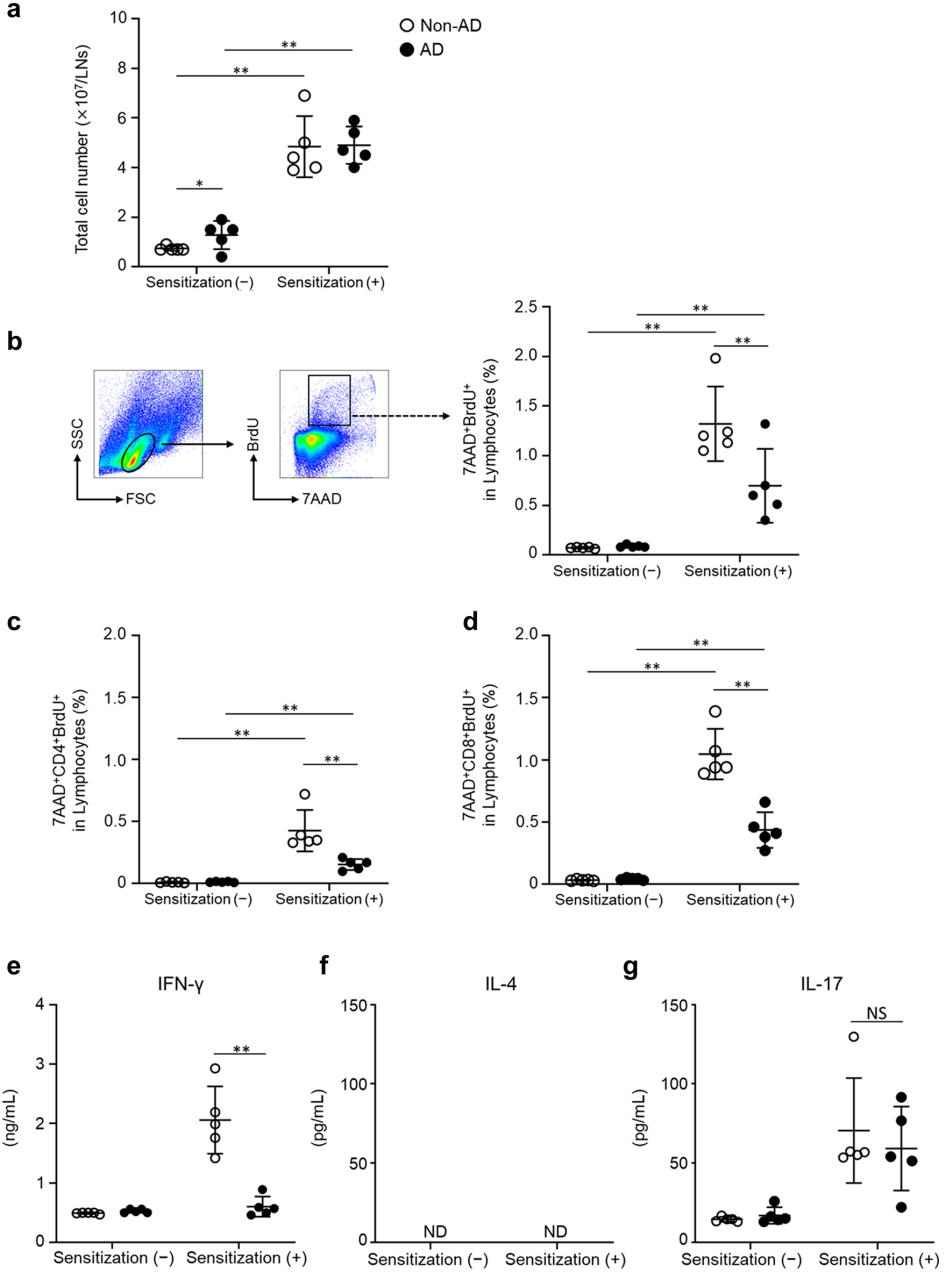
Impaired DNFB-specific T cell proliferation and cytokine production after DNFB sensitization in AD mice. Draining LNs were collected five days after sensitization of non-AD and AD mice with or without 0.3% DNFB. (**a**) The total number of draining LN cells. (**b–g**) DNBS-induced lymphocyte proliferation and cytokine production. Draining LN cells were collected from non-AD and AD mice five days after 0.3% DNFB application and cultured for two days with or without 50 µg/mL DNBS. Cell proliferation was evaluated using the BrdU assay. (**b**) Representative flow cytometry dot plots (left) and the frequency of 7AAD^+^BrdU^+^ cells per lymphocyte with DNBS. (**c**) 7AAD^+^CD4^+^BrdU^+^ cells, (**d**) 7AAD^+^CD8^+^BrdU^+^ cells per lymphocyte with DNBS. (**e–g**) The amount of IFN-γ, IL-4, and IL-17 in the culture medium was measured by ELISA. Data were expressed the mean values ± *SD* (n = 5) and represented two independent experiments with similar results. *p < 0.05, **p < 0.01 between indicated groups, ND; not detected, NS; not significant.

To evaluate whether T cell proliferation and cytokine production are impaired in AD mice after DNFB sensitization, the draining lymph node (LN) cells from non-AD and AD mice were challenged with or without DNBS in vitro. We collected draining LNs from mice five days after 0.3% DNFB sensitization. Then, we examined the total number of draining LN cells before re-stimulation with DNBS, a water-soluble compound with the same antigenic properties as DNFB. In the non-sensitized mice, the total number of draining LN cells was significantly higher (*p* = 0.024) in AD than in non-AD mice, indicating AD-induced cell proliferation may occur in AD mice. On the other hand, in the DNFB-sensitized mice, the number of draining LN cells in non-AD and AD mice was comparable, indicating increased cell proliferation by DNFB sensitization in non-AD was higher than in AD mice (Fig. 2a).

Subsequently, we measured effector T cell proliferation induced by DNBS using the Bromodeoxyuridine (BrdU) assay. The results of re-stimulation with DNBS showed that DNFB sensitization significantly increased (non-AD; *p* = 0.00007, AD; *p* = 0.00005) the percentage of 7AAD^+^BrdU^+^cells in draining LN cells in non-AD and AD mice compared to the non-sensitized group. In the sensitized group, the percentage of BrdU^+^cells was significantly decreased (*p* = 0.002) in AD mice compared to non-AD mice (Fig. 2b and Suppl. Figure 1a). Moreover, the percentage of 7AAD^+^CD4^+^BrdU^+^ and 7AAD^+^CD8^+^BrdU^+^ cells in lymphocytes with DNBS re-stimulation was significantly lower (7AAD^+^CD4^+^BrdU^+^; *p* = 0.008, 7AAD^+^CD8^+^BrdU^+^; *p* = 0.001) in DNFB-sensitized AD than that in DNFB-sensitized non-AD mice (Fig. 2c,d and Supplementary Fig. 1b).

Furthermore, the production of IFN-γ induced by the DNBS re-stimulation in the culture supernatant was markedly decreased (*p* = 0.0006) in draining LNs from DNFB-sensitized AD mice compared with those from DNFB-sensitized non-AD mice (Fig. 2e). IL-4 production was not detected in either AD or non-AD mice, or IL-17 production was not different between AD and non-AD mice (Fig. 2f,g). Therefore, hapten-specific T cell proliferation and IFN-γ production were suppressed during sensitization in AD mice.

### Frequency of CTLA-4^+^Treg cells was higher in AD mice

**Figure 3:**
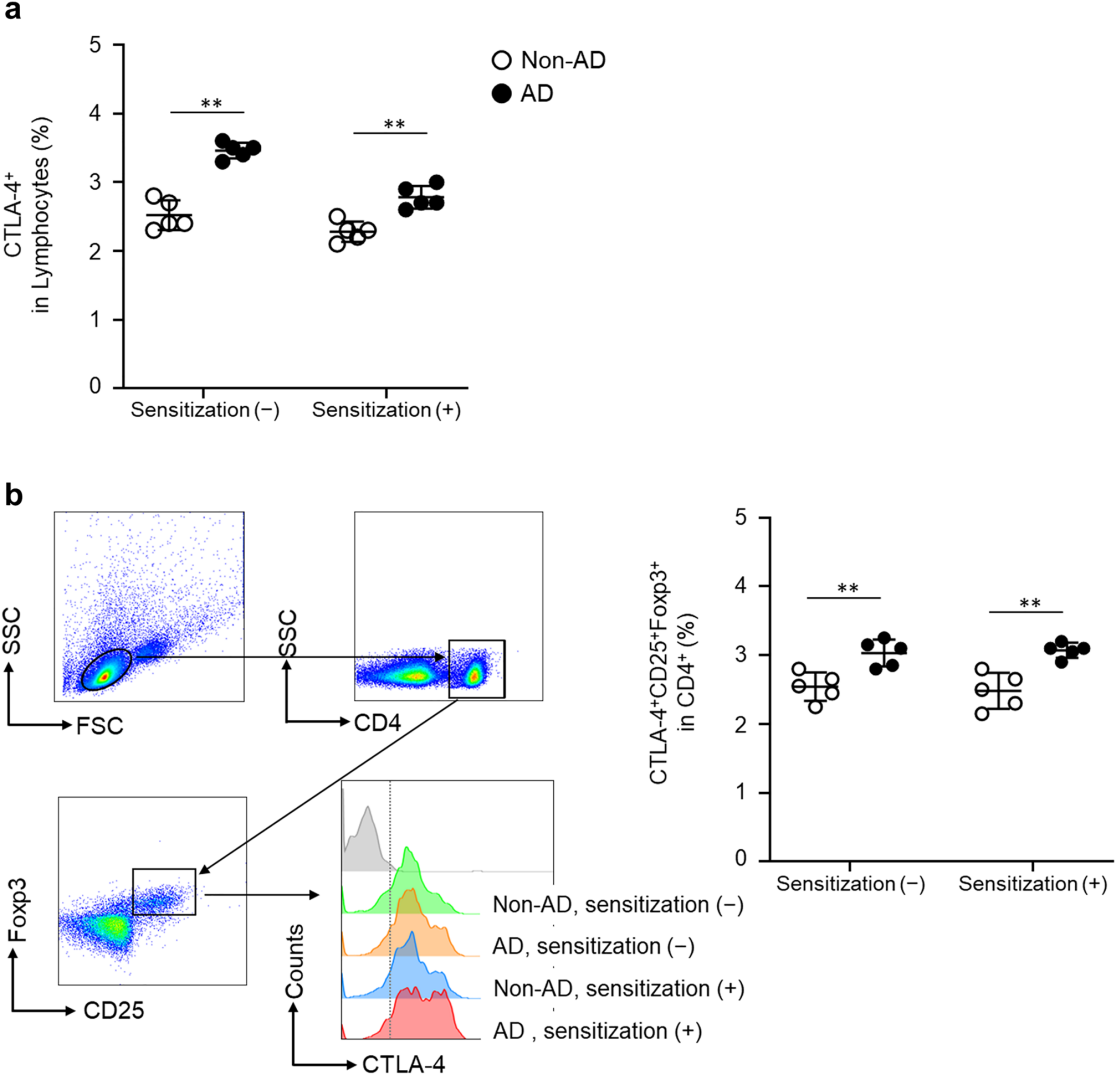
CTLA-4^+^Treg cells increased in AD mice. Draining LNs were collected from non-AD and AD mice one day after 0.3% DNFB sensitization. (**a**) CTLA-4^+^cells per lymphocyte (**b**) Representative flow cytometry histogram results (left) and the frequency of CTLA-4^+^CD25^+^FoxP3^+^Treg cells per CD4^+^. Data were expressed as the mean values ± *SD* (n = 5) and represented two independent experiments with similar results. *p < 0.05, **p < 0.01 between indicated groups.

Next, we explored the mechanisms for the suppression of CHS responses in AD mice. CTLA-4^+^T cells may regulate the hapten-specific T cell proliferation during the CHS response^10^. Thus, we analyzed the CTLA-4^+^T cells in draining LNs after DNFB sensitization. The percentage of CTLA-4^+^T cells in lymphocytes on day one after 0.3% DNFB sensitization was higher (non-sensitization; *p* = 0.002, sensitization; *p* = 0.027) in AD mice in both non-sensitized and sensitized mice (Fig. 3a). CTLA-4 expressed on Treg cells can suppress T cell activation^16^. Therefore, we examined the percentage of CTLA-4 expressing CD4^+^CD25^+^Foxp3^+^cells (Treg cells) showed that the percentage of CTLA-4^+^Treg cells was significantly higher (non-sensitization; *p* = 0.040, sensitization; *p* = 0.044) in AD mice (Fig. 3b). In this experiment, we simply focused on CTLA-4 expressing Treg cells, not a population of Treg cells that strongly express CTLA-4, as shown in the histogram in Fig. 3b. Taken together, CTLA-4^+^Treg cells may contribute to the attenuated CHS response in AD mice.

### CTLA-4 contributes to the attenuation of CHS response in AD mice

**Figure 4:**
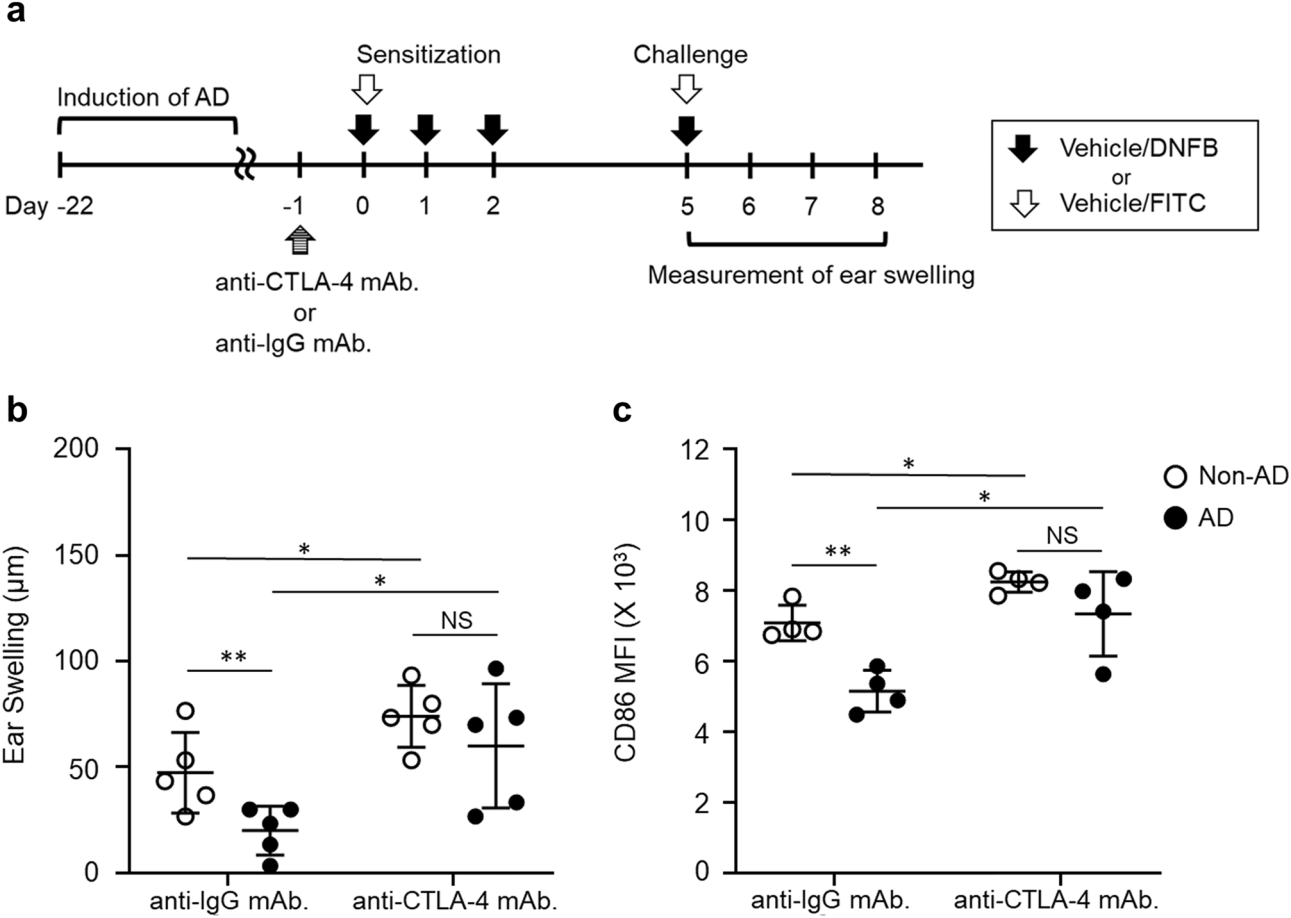
Ear swelling and expression of CD86 on migratory DCs between non-AD and AD mice are comparable after treatment with anti-CTLA-4 mAb. Mice were treated with anti-CTLA-4 mAb or control anti-IgG mAb 1 day before 0.3% DNFB or 1.0% FITC sensitization. (**a**) Treatment of mice was performed as per the schematic. (**b**) Ear swelling was measured after the challenge with 0.3% DNFB. Data represents the change in ear thickness at 24 h. (**c**) Mice were sensitized to FITC and the expression level of CD86 on MHC class II^high^FITC^+^migratory DCs. Data were expressed as mean ± *SD* (n = 5) and represented two independent experiments with similar results. *p < 0.05, **p < 0.01 between the indicated groups.

To clarify the contribution of CTLA-4^+^T cells to suppressing the CHS response in AD mice, we investigated the effects of CTLA-4 blockade on the CHS responses. Both non-AD and AD mice were administered with either the control anti-IgG mAb or anti-CTLA-4 mAb one day before 0.3% DNFB sensitization (Fig. 4a). Both non-AD and AD mice showed that ear swelling at 24 h was enhanced (non-AD; *p* = 0.038, AD; *p* = 0.022) in the anti-CTLA-4 mAb group compared to the anti-IgG mAb group after the challenge. Furthermore, in the anti-IgG mAb group, the CHS response was attenuated (*p* = 0.025) in AD mice compared to non-AD mice, which was a similar result as in the untreated antibody (Fig. 1c). On the other hand, no difference was observed between non-AD and AD mice of the anti-CTLA-4 mAb group (Fig. 4b and Supplementary Fig. 2).

CTLA-4 competes with CD28 to bind to CD80/CD86 on DCs, which results in the downregulation of CD80/CD86 expression to inhibit T cell activation^17^. To investigate the contribution of CTLA-4 to DCs, we examined the effect of CTLA-4 blockade on the CD86 expression in DCs. We analyzed the expression levels of CD86 in MHC class II^high^FITC^+^ migratory DCs using fluorescein isothiocyanate (FITC), which is typically employed to identify cutaneous migratory DCs in draining LNs after sensitization^18^. Administration of anti-CTLA-4 mAb significantly increased (non-AD; *p* = 0.007, AD; *p* = 0.017) the CD86 expression on MHC class II^high^FITC^+^ migratory DCs on day one after FITC sensitization in both non-AD and AD mice. In the anti-IgG mAb group, CD86 expression was significantly decreased (*p* = 0.003) in AD mice compared to non-AD mice, whereas in the anti-CTLA-4 mAb group, CD86 expression showed no significant difference between non-AD and AD mice, and CD86 expression was restored in AD mice to a similar level as non-AD mice by administration of anti-CTLA-4 mAb (Fig. 4c). These results suggest that CTLA-4 suppresses the CHS response in AD mice by downregulating CD86 expression on migratory DCs after sensitization.

## Discussion

We investigated the CHS response to the strong sensitizer DNFB in AD-induced NC/Nga model mice and found that the inflammatory response determined by ear swelling was reduced in AD mice compared to non-AD mice. Hapten-specific T cell proliferation and IFN-γ production were significantly lower in draining LNs of AD compared to non-AD mice, and these results reflected clinical experiments. Furthermore, the percentage of CTLA-4^+^T cells was higher in AD mice regardless of sensitization. CHS response differences observed between non-AD and AD mice in the antibody untreated group were eliminated by the anti-CTLA-4 mAb experiment. These results suggest that a suppressive mechanism generated by CTLA-4^+^T cells may contribute to the attenuated CHS response in AD mice.

Previous reports examined the CHS response in the filaggrin-deficient flaky tail (ft/ft) and FlgHrnr double-deficient (FlgHrnr^−/−^) mice, which is a model that reflects the skin barrier status of AD and showed that the CHS response was exacerbated in AD mice compared to controls^13,19^. Although these results conflict with our findings, differences in the immune status among AD mice may account for the discrepancies in the results^20,21^. As with AD in human, AD-induced NC/Nga mice showed type 2 cytokine activation^22^ and increased frequency of Treg cells (Fig. 3b), but these differences in the activation of Th2 cells or Treg cells may have influenced the results among AD models.

A previous report showed that CTLA-4^+^T cells contribute to suppressing the CHS response^10^. Among CTLA-4 expressed T cells, Treg cells can regulate the CD80/86 expression in DCs during the CHS responses^23,24^. Moreover, depletion of Treg cells exacerbates the CHS response (i.e., expansion of T cell proliferation and IFN-γ production)^25^. CTLA4-mediated depletion of CD80 and CD86 from APCs results in reduced T cell activation due to impaired CD28 co-stimulation^26^. In this study, we found that the percentage of CTLA-4^+^Treg cells was higher in AD mice (Fig. 3b) and that CTLA-4 and CD86 expression on migratory DCs are involved in the suppressive mechanism of the CHS response in AD, based on the experiments with anti-CTLA-4 mAb administration (Fig. 4). These results suggest that CTLA-4 downregulated the CD86 expression on migratory DCs after hapten sensitization in AD mice, and that hapten-specific T cell proliferation, IFN-γ production and CHS responses were suppressed.

The percentage of CTLA-4^+^T cells and CTLA-4^+^Treg cells can be higher in AD patients than in healthy controls^11,12^, which is consistent with the findings in NC/Nga mice in the present study. The higher percentage of CTLA-4^+^T cells in AD mice before and after sensitization suggests that CTLA-4^+^T cells, which are not hapten-specific but increased with the onset of AD, may contribute to the suppression mechanism of the CHS response. Therefore, the suppression mechanism of ACD response by CTLA-4^+^T cells may be active in AD patients.

Like in AD patients, hapten-specific Th1 cell proliferation and CHS response were suppressed in DNFB-sensitized AD mice. However, it is unclear whether similar results could be obtained using other sensitizers. Therefore, in addition to DNFB, an experimental allergen, other allergens, such as common allergens, should be investigated to confirm the findings.

In this study, we found that the CHS response to DNFB in AD mice was attenuated compared to non-AD mice, reflecting clinical experiments. Furthermore, the suppression mechanism by CTLA-4 may contribute to the attenuated CHS response in AD mice, as suggested by experiments with anti-CTLA-4 mAb. Therefore, CTLA-4^+^T cells may contribute to the attenuated ACD response to strong sensitizers in AD patients. However, further study is required to examine the universality of different allergens.

## Methods

### Animals

Male NC/Nga mice were purchased from Japan SLC (Shizuoka, Japan) and used in experiments at 8–10 weeks of age. Mice were maintained at Kao Corporation facility at 22 °C ± 1 °C room temperature, 40–60% humidity, on a 12 h light–dark cycle (7 a.m. to 7 p.m.), and given food and water ad libitum, according to institutional guidelines. All experiments were performed under inhalation anesthesia with isoflurane (Pfizer Inc., USA), and mice were euthanized by cervical dislocation at the end of the experiment. All animal experimental protocols were reviewed and approved by the Kao Corporation Animal Care Committee, and all methods were conducted in accordance with the guidelines of the Kao Corporation Animal Care Committee. We conducted all animal experiments in accordance with ARRIVE guidelines.

### Induction of AD mouse model

AD-like skin lesions in NC/Nga mice were induced by Biostir-AD (Biostir Inc., Osaka, Japan), an extract of the house dust mite *Dermatophagoides farina*. The manufacturer's instructions and method reported in a previous report^27^ were modified to use in this study. At first, 100 mg of Biostir-AD was applied to the shaved dorsal skin and both ears. For the second application, 150 µL of 4% sodium dodecyl sulfate solution was applied to the shaved dorsal skin (100 µL) and both ears (25 µL each per ear). After drying for 3 h, 100 mg of Biostir-AD was applied to the back skin and both ears. While both ears were treated with Biostir-AD only for the first and second applications, the application of Biostir-AD onto the dorsal skin was repeated twice a week for 3 weeks.

### Measurement of skin severity score

TEWL and dermatitis severity was assessed to verify the skin lesion condition^28^. The clinical severity of skin lesions was scored as 0 (none), 1 (mild), 2 (moderate), or 3 (severe) for developing erythema hemorrhage, scar dryness, edema, and exfoliative erosion. The sum of the individual scores was used as the dermatitis score. AD mice were categorized between control and hapten-sensitized mice with similar average clinical skin scores.

### DNFB-induced CHS model

DNFB-sensitized mice were generated as previously described^29,30^. Mice were sensitized by applying 20 µL of 0.3% DNFB (v/v) (Sigma-Aldrich, St. Louis, MO, USA) dissolved in acetone/olive oil (4:1, v/v) (AOO) on the shaved dorsal skin on days 0, 1, and 2. The DNFB concentration was prepared at 0.003%, 0.03%, or 0.3%, and mice were sensitized with each concentration of DNFB (Fig. 1d). The control mice were treated with AOO as a vehicle. Five days after sensitization, mice were challenged with 10 µL of 0.3% DNFB applied to each side of the right ear.

### Measurement of the ear thickness

Ear thickness was measured using a digital thickness gauge (Mitutoyo, Japan) before, at 24, 48, 72 and 92 h after the challenge. The ear swelling was calculated as [(*T–T*_0_ right ear)] − [(*T–T*_0_ left ear)], where *T*_0_ and *T* were the values of ear thickness before and after the challenge, respectively.

### T cell proliferation assay and cytokine production

RPMI 1640 medium containing 10% heat-inactivated fetal calf serum, 2 mM l-glutamine, 50 µM 2-mercaptoethanol, 25 mM *N*-2-hydroxyethyl piperazine-*N* 9-2-ethane sulfonic acid (HEPES), 1 × MEM non-essential amino acid solution, 100 units/mL penicillin and 100 μg/mL streptomycin purchased from Thermo Fisher Scientific were used as culture medium.

Single-cell suspensions were prepared from draining LNs of mice on day 5 after DNFB sensitization. Referring to previous reports^31,32^, 5 × 10^5^ LN cells were stimulated with or without 50 µg/mL sodium DNBS (Sigma-Aldrich, St. Louis, MO, USA) for 48 h, and BrdU was added for the last 12 h. T cell proliferation analysis protocol was performed using the BrdU Flow Kit (BD Bioscience) according to the manufacturer’s instructions. Cell suspensions were Fc blocked with CD16/CD32 (2.4G2, BD Bioscience) and stained with anti-CD4 (GK1.5), anti-CD8 (53–6.7), and 7-aminoactinomycin D (7-AAD) purchased from BD Bioscience.

To measure the cytokine production, culture supernatants were collected 48 h post-induction. The amount of IFN-γ, IL-4, and IL-17 in the culture supernatants was measured using ELISA Kit (R&D system) following the manufacturer’s instructions.

### Analysis of Treg cells

CTLA-4^+^Treg cells were analyzed as previously described^33^. Flow cytometric experiments were conducted on draining LN cells collected from the axillary/inguinal LNs one day after sensitization. Cell suspensions were Fc blocked with CD16/CD32 (2.4G2, BD Bioscience) and stained with the following anti-mouse antibodies purchased from BD Bioscience: anti-CD4 (GK1.5), anti-CD8 (53–6.7), anti-CD25 (PC61) and anti-CTLA-4 (UC10-4F-11). Intracellular staining with anti-FoxP3 (MF23) and anti-CTLA-4 (UC10-4F-11) was performed using the FoxP3/Transcription Factor Staining Buffer Set (eBioscience). FACS analysis was conducted using a FACS Lyric flow cytometer (BD Bioscience), and data were generated using FlowJo software (Tree star, Ashland, OR).

### Administration of CTLA-4 blocking monoclonal antibody

Treatment with antibody administration was performed as previously described with modifications^10^. Mice were intraperitoneally (i.p) injected with 500 µg/mouse of the anti-mouse CTLA-4 monoclonal antibody (mAb) (9D9, BioXcell) or 500 µg/mouse of the anti-mouse IgG2b mAb (MPC-11, BioXcell) isotype control one day before 0.3% DNFB or 1.0% FITC sensitization (day^−1^).

### Analysis of migratory DCs

Mice were sensitized with 150 µL of 1.0% FITC (DOJINDO, Kumamoto, Japan) in acetone/dibutyl phthalate (1:1, v/v) was applied on shaved dorsal skin at day 0.

Migratory DCs were analyzed as previously described^34^. Flow cytometric experiments were conducted on draining LN cells collected from the axillary/inguinal LNs one day after sensitization. For single-cell suspensions, preparation from the draining LNs was digested with collagenase type I (Sigma-Aldrich, 400 U/L) at 37 °C for 30 min and incubated with ethylenediaminetetraacetic acid (EDTA, final concentration 5 mM) for an additional 10 min. Cell suspensions were Fc blocked with CD16/CD32 (2.4G2, BD Bioscience) and stained with the following anti-mouse antibodies purchased from BD Bioscience: anti-MHC class II (M5/114.15.2) and anti-CD86 (GL1). Dead cells were labeled with 7-AAD (BD Biosciences).

### Statistical analysis

Significant differences between experimental groups were analyzed using Student’s t-test. *P* values < 0.05 were considered statistically significant.

## Supplementary Information

Supplementary Figures.

## Data Availability

The datasets generated and/or analyzed during the current study are available from the corresponding author upon reasonable request.
