# Author Correction: CTLA-4 suppresses hapten-induced contact hypersensitivity in atopic dermatitis model mice

**DOI:** 10.1038/s41598-023-37446-w

**Published:** 2023-06-29

**Authors:** Hiroe Tetsu, Kanako Nakayama, Taku Nishijo, Takuo Yuki, Masaaki Miyazawa

**Affiliations:** Safety Science Research Laboratories, Kao Corporation, 2606, Akabane, Ichikai, Haga, Tochigi 321-3497 Japan

Correction to: *Scientific Reports* 10.1038/s41598-023-35139-y, published online 16 May 2023

The original version of this Article contained errors in the name of author Takuo Yuki, which was incorrectly given as Yuki Takuo.

The original Article and accompanying Supplementary Information file have been corrected.

