# Supplementary material for: CTLA-4 suppresses hapten-induced contact hypersensitivity in atopic dermatitis model mice
Source: Sci Rep. 2023 May 16;13:7936. doi: 10.1038/s41598-023-35139-y (PMC10188484; doi:10.1038/s41598-023-35139-y)
Supplement: Supplementary file 1 — Supplementary Figures. [file 41598_2023_35139_MOESM1_ESM.pdf]

Supplementary Information file

CTLA-4 Suppresses Hapten-Induced Contact Hypersensitivity in Atopic Dermatitis Model Mice

Hiroe Tetsu[1*], Kanako Nakayama[1], Taku Nishijo[1], Takuo Yuki[1], Masaaki Miyazawa[1]

[1]Safety Science Research Laboratories, Kao Corporation, 2606 Akabane, Ichikai, Haga, Tochigi, 321-3497, JAPAN

*Corresponding author: Hiroe Tetsu (tetsu.hiroe@kao.com)

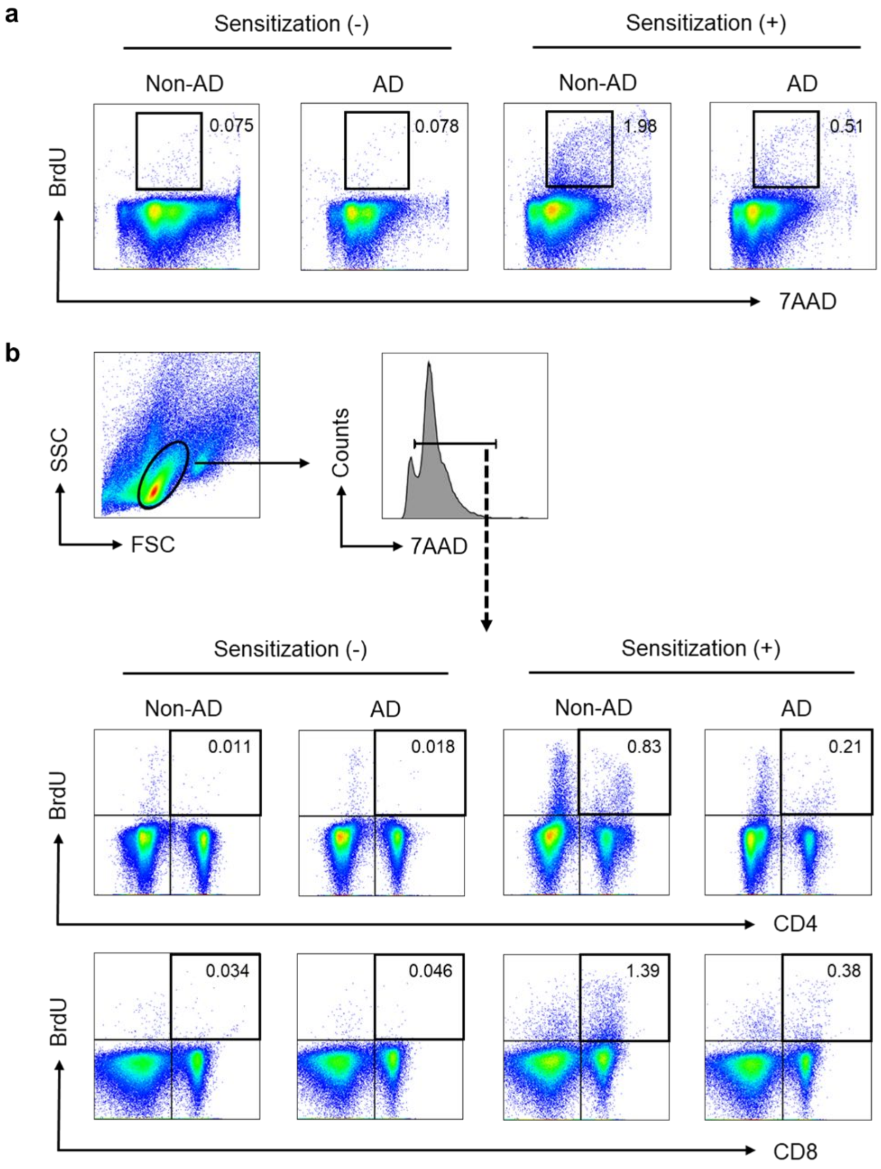

Supplementary Figure 1. Flow cytometry analysis and gating strategy for identification of DNFB-specific T cell proliferation.

This figure shows the flow cytometry dot plots corresponding to the results of Figure 2b-d. Representative flow cytometry dot plots showing the frequency of the percentage of (a) $7AAD^{+}BrdU^{+}$ cells, (b) $7AAD^{+}CD4^{+}BrdU^{+}$ cells and $7AAD^{+}CD8^{+}BrdU^{+}$ cells per lymphocytes with DNBS. Data were expressed as mean ±*SD* (n = 5) and represented two independent experiments with similar results.

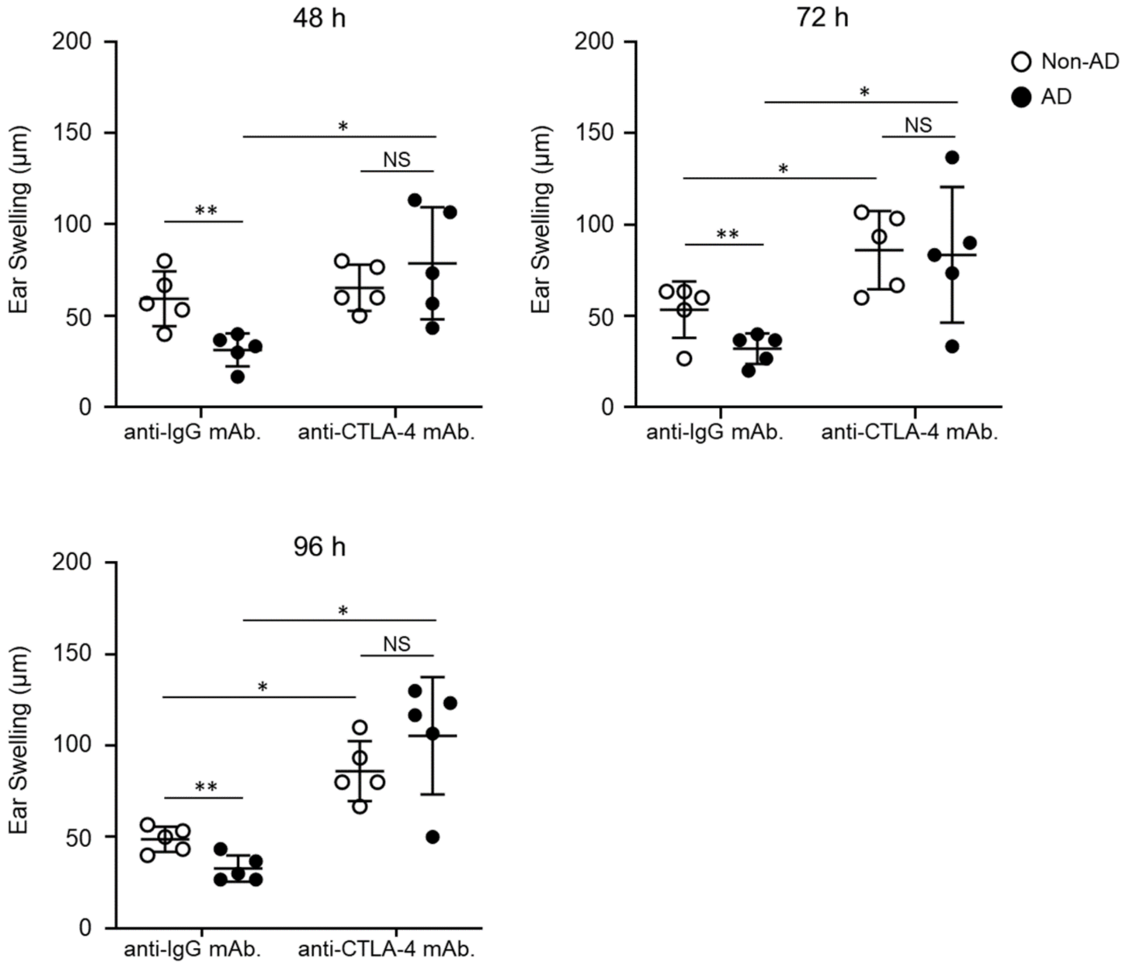

Supplementary Figure 2. Time course changes of ear swelling after administration of anti-CTLA-4 mAb.

This figure represents the same data as in Figure 4b, but at a different measurement time. Mice were treated with anti-CTLA-4 mAb or control anti-IgG mAb one day before 0.3% DNFB. Ear swelling was measured after the challenge with 0.3% DNFB sensitization. Data represents the change in ear thickness at 48, 72 and 96h (Fig. 4b shows data for 24h). Data were expressed as mean ±*SD* (n = 5) and represented two independent experiments with similar results. *$p < 0.05$, **$p < 0.01$ between the indicated groups.
